# Sex and age-related patterns in pediatric primary headaches: observations from an outpatient headache clinic

**DOI:** 10.3389/fneur.2024.1441129

**Published:** 2024-08-19

**Authors:** Vanda Faria, Berit Höfer, Anna Klimova, Maja von der Hagen, Reinhard Berner, Rainer Sabatowski, Thea Koch, Anke Hübler, Matthias Richter, Eric A. Moulton, Scott A. Holmes, Gudrun Gossrau

**Affiliations:** ^1^Department of Psychology, Uppsala University, Uppsala, Sweden; ^2^Pain and Affective Neuroscience Center, Department of Anesthesia, Critical Care and Pain Medicine, Boston Children's Hospital, Harvard Medical School, Boston, MA, United States; ^3^Comprehensive Pain Center, Faculty of Medicine and University Hospital Carl Gustav Carus, TUD Dresden University of Technology, Dresden, Germany; ^4^NCT Partner Site Dresden, Institute for Medical Informatics and Biometrics, Faculty of Medicine Carl Gustav Carus, TU Dresden, Dresden, Germany; ^5^Abteilung für Neuropädiatrie, Medizinische Fakultät Carl Gustav Carus, Technische Universität Dresden, Dresden, Germany; ^6^Department of Pediatrics, University Hospital and Faculty of Medicine Carl Gustav Carus, TU Dresden, Dresden, Germany; ^7^Department of Anesthesiology and Intensive Care, University Hospital and Faculty of Medicine Carl Gustav Carus, TU Dresden, Dresden, Germany; ^8^Brain and Eye Pain Imaging Lab, Department of Anesthesia, Critical Care and Pain Medicine, Boston Children's Hospital, Harvard Medical School, Boston, MA, United States; ^9^Department of Ophthalmology, Department of Anesthesia, Critical Care and Pain Medicine, Boston Children's Hospital, Boston, MA, United States

**Keywords:** migraine, pediatric headache, primary headache, tension-type headache, sex, age, puberty, girls

## Abstract

**Background:**

Age reportedly affects headache prevalence differently in boys and girls. However, little empirical data exists regarding pediatric headache prevalence and headache-related burden in children and adolescents according to age and sex. In the present study, we considered age and sex while evaluating the distribution, characteristics, and impairment of primary headache disorders at a pediatric headache center in Germany.

**Methods:**

Medical records of children and adolescents attending the headache clinic of the Interdisciplinary Pain Center of the Carl Gustav Carus University Hospital in Dresden during the period 2015–2022 were retrospectively grouped and analyzed depending on age (< or ≥14 years) and sex.

**Results:**

The study population consisted of 652 children and adolescents, aged between 3 and 18 years. Almost two-thirds of the patients (≈60%) were females, and almost two-thirds of these females (58%) were ≥14 years of age. Generally, the most prevalent headache diagnoses as defined by the International Classification of Headache Disorders 3rd edition were episodic migraine without aura and the combination of tension-type headache and episodic migraine with or without aura i.e., mixed-type headache (each ≈27%). In the younger group (<14 years), the mixed-type headache was the most prevalent in girls (28.6%), whereas, for boys, episodic migraine without aura was the most prevalent headache diagnosis (47.4%). In the older group (≥14 years), the mixed-type headache continued to be the most prevalent for girls (30%), and it became the most prevalent for boys (26.3%). Before the age of 14, about 16% of children were severely affected by their headaches. After the age of 14, this proportion increased to roughly one-third (33%) of adolescents, driven mainly by teenage girls (26%) who were severely affected by their headaches. Furthermore, the prevalence of comorbidities was significantly higher among girls (67%), particularly in the adolescent group (74%).

**Conclusions:**

Our data shows that headache disorders in a specialized pediatric clinic impose a significant burden, especially among teenage girls indicating high therapy needs. Enhancing awareness of early diagnosis and preventive care is crucial to mitigate the development of chronic headaches, and mitigate their adverse effects on life quality and educational capability.

## Introduction

Primary headache disorders are a major public health concern. According to the latest Global Burden of Disease study, headache disorders remain the top third leading cause of years lived with disability worldwide (1–3). In adults, the overall prevalence of headache is estimated between 46 and 79%, for tension-type headache 38 and 42%, for migraine 11 and 35%, and probable medication overuse headache 3.1% (4). The reported high prevalence in adults, plus the headache-related loss of daily activities and productivity, the development of comorbid disorders, and an early onset of the headache disease increase the overall burden of headache disorders (5). Even though the burden of headaches is not as well-documented in children as compared to adults (6, 7), pediatric headache disorders have the added potential effect of impacting education and social functioning during decisive developmental years that will most likely impose an increased burden throughout life for both individuals and society at large (8).

In pediatrics, headache is the most common neurological symptom and the most common reported pain (9). The estimated lifetime prevalence of overall primary headaches in children is about 62% with prevalence in females and males of 38 and 27%, respectively (10), and with up to 47% of children and adolescents reporting recurrent headaches (11). The prevalence of tension-type headache varies between 58% in Norway (12) and 6.2% in Kuwait (7) depending on the age of investigated children and other methodological differences. Concerning migraine, prevalence between 23 and 10.9% are reported (7, 12). A recent meta-analysis suggested a global prevalence of 11% for migraine overall, 8% for migraine without aura, 3% for migraine with aura, and 17% for tension-type headache (10). The prevalence of primary headaches increases throughout childhood (13), and by the age of 18 years, 90% of adolescents have had a headache (14). Whereas, before puberty headache prevalence is similar between boys and girls, after puberty, headache disorders become significantly more prevalent in girls. This difference is greatly determined by an increased prevalence of migraine in females after puberty (female 12–17%; male 4–6%), which then display a ratio similar to what is known from adult studies (10), highlighting the potential role of sex hormones in headache pathophysiology but likely sociocultural factors as well (15).

Migraine is thought to be a cyclic disorder with a complex sequence of symptoms, which can vary with age. The overall clinical presentation of migraine in children as compared to adults consists of shorter, mainly bilateral attacks, and a display of vegetative gastro-intestinal and non-nociceptive symptoms, the latter characterized as migraine variants (6). Age-dependent differences in headache clinical symptoms seem to be less prominent in tension-type headache (16). However, tension-type headache symptoms in children are often a mixed headache with migraine and the differentiation can be challenging (17). Even though specific pediatric criteria have been included in the headache diagnostic criteria (ICHD-3) (18), symptom variability in children of different ages and sex may lead to classification difficulties. Classifying pediatric headache disorders, especially but not only in younger children, has additional clinical challenges that possibly underlie the lack of specific diagnostic markers which may result in underdiagnosed, underestimated, and most likely untreated pediatric headache disorders.

Accumulating evidence suggests an increasing prevalence of pediatric headaches (19), that are arguably responsible for a remarkable impact on physical and psychological wellbeing (20). However, the impairment caused by headache disorders in the pediatric population is still poorly understood, particularly the differential impact of puberty on headache prevalence in boys and girls. The goal of this study is to provide a comprehensive description of the distribution, characteristics, and impairment of everyday function of pediatric primary headache disorders according to age and sex in a headache outpatient clinic in Germany. Prospectively, this can be used to address specific public and individual interventions for awareness, education, and management of headaches in children and adolescents.

## Methods

In this monocentric study, medical records of children and adolescents who attended the headache outpatient clinic at the interdisciplinary pain center of the Carl Gustav Carus University Hospital in Dresden from September 2015 to July 2022 were retrospectively reviewed. Headache diagnoses and classifications were performed according to the International Classification of Headache Disorders, Third Edition (ICHD-3) by a pediatrician (MR) and pain therapist (AH), experienced in pediatric headache diagnosis and treatment under supervision of a neurologist (GG), specialized in headache (18). During their visits to the Headache clinic, all patients and their parents were interviewed using a format based on the ICHD-3 criteria (18), in addition personal and family medical history, and were requested to complete a questionnaire (PedMIDAS) and demographic questions as published elsewhere (13). In addition, the headache diary provided by the German Society for Migraine and Headache was used, where children and adolescents entered their headache days, intensity (according to a numerical analog scale), and accompanying symptoms such as i.e., nausea, aura, or photophobia (English version of the diary shown here: https://www.dmkg.de/files/Kopfschmerzkalender_PDF/Kopfschmerzkalender_ENGLISCH_18.3.2021.pdf).

### Headache diagnoses

Based on ICHD-3 criteria (18), headache disorders were divided into seven groups consisting of (1) Episodic migraine with aura; (2) Episodic migraine without aura; (3) Chronic migraine; (4) Episodic Tension-type headache; (5) Chronic tension-type headache; (6) Migraine and tension-type headache mixed i.e., Mixed-type headache (any form of tension-type headache and episodic migraine with or without aura); (7) Other primary headaches (e.g., new daily persistent headache, vestibular migraine, trigeminal autonomic headaches). Headaches of included patients were carefully assessed and patients with secondary headaches were not included in this study. Only in one case, a patient with a secondary headache due to a craniopharyngioma was included since the secondary headache resolved after appropriate tumor treatment and the same patient experienced migraine attacks.

### Comorbidities

The comorbidities commonly encountered in primary headache disorders were assessed and allocated into six main disease groups, based on the affected biological system:

1. Internal, ear, and ophthalmological diseases: hypopituitarism, von Willebrand-Syndrome, essential hypertension, bronchial asthma, cystic renal dysplasia, obesity, irritable bowel syndrome, tuberculosis, adrenogenital syndrome, dilatative uropathy, non-Hodgins lymphoma, iron deficiency, autoimmune thyroiditis, Vit. D-hypovitaminosis, dysganglionosis, fructose malabsorption, cirrhosis of the kidney, hyperthyroidism, conductive hearing loss, and chronic papillitis.
2. Mental disorders: depression, obsessive-compulsive disorder, bulimia, post-traumatic stress disorder, adjustment disorder, specific phobia, fatigue syndrome, attention deficit disorder (ADS), and autism.
3. Orthopedic diseases: scoliosis, cervical spine joint blockage, craniomandibular dysfunction.
4. Neurological diseases: epilepsy, craniopharyngioma, and arachnoidal cyst as incidental finding without pathological value.
5. Dermatological diseases: neurodermitis, urticaria factitia.
6. Chronic pain syndromes: cervical neuralgia, chronic pain syndrome involving psychological and somatic factors, abdominal pain, musculoskeletal pain amplification syndrome, low back pain, intermittent knee pain, and otalgia.

### Statistical analysis

Statistical analyses were performed using SPSS software version 29 (SPSS, Chicago, IL, USA). Categorical variables were defined as frequencies and percentages and continuous variables were expressed as means and standard deviations or medians (with interquartile range). To compare groups, Chi-square tests, *t*-tests, and the Mann-Whitney *U*-test were used as appropriate.

## Results

### Study population and characteristics

**Figure 1:**
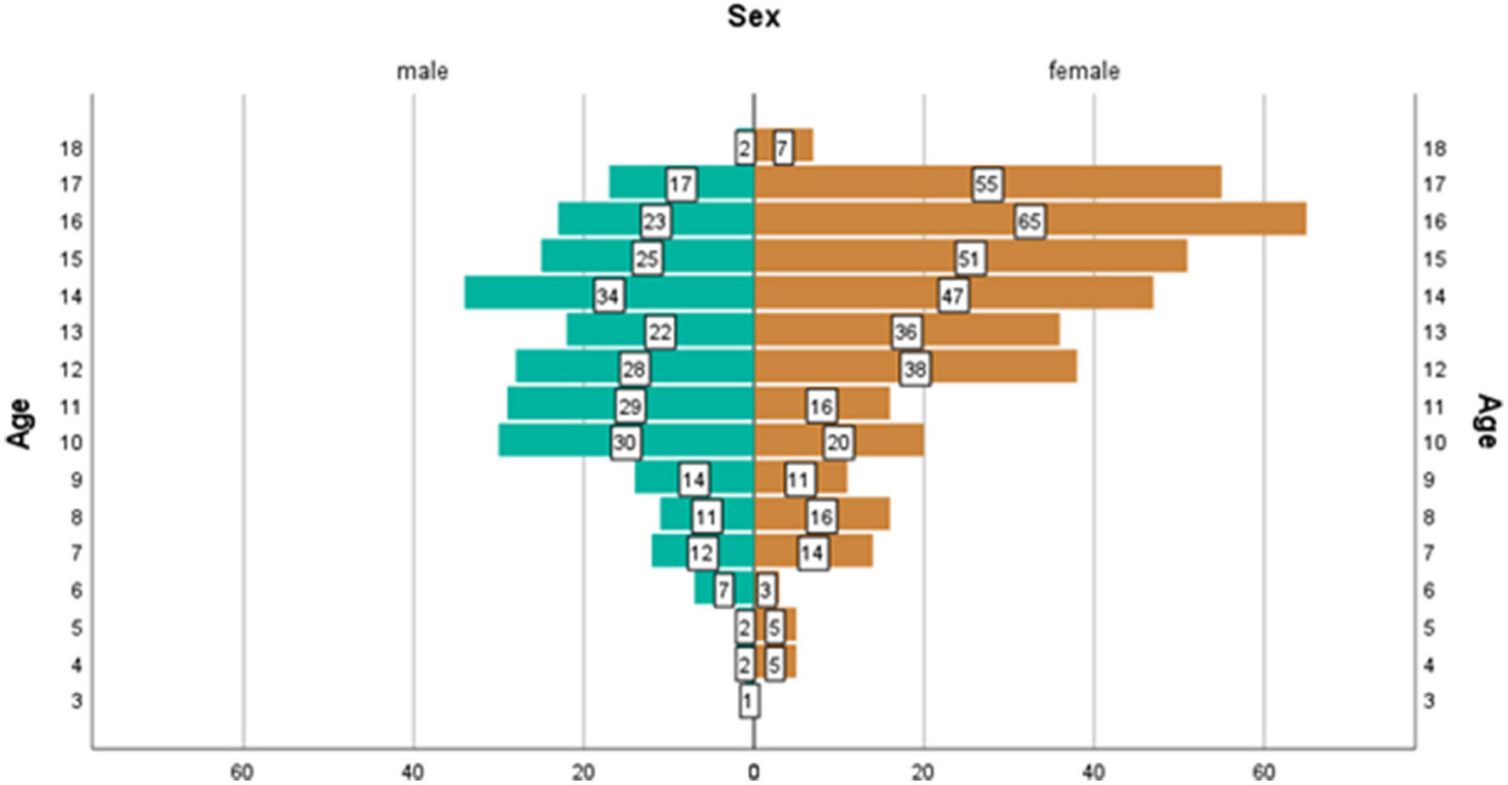
Age by sex distribution of children with primary headache disorders (*n* = 652).

Our sample was composed of 652 children with headaches, aged between 3 and 18 years (age, mean ± SD, 12.9 ± 3.3 years). Girls represented 59.8% (*n* = 390) (age, mean ± SD, 13.4 ± 3.2 years) and boys represented 40.2% (*n* = 262) (age, mean ± SD, 12.2 ± 3.2 years) of the entire sample. Girls were significantly overrepresented (χ^2^ = 25.13, df = 1, *p* < 0.001) and were significantly older than the boys (*t* = 4.88, df = 646, *p* < 0.001). Age by sex distribution in children with primary headache disorders (Figure 1), shows that almost two-thirds of the girls (225; 58%) were older than 13 years, whereas almost two-thirds of the boys (158; 61%) were younger than 14 years. Based on the characteristics of our sample and previous reports of health insurance data in Germany from ~1.2 million children and adolescents, suggesting that pediatric incidence of migraine in boys and girls is similar up to age 13, with a significant increase in incidence in girls from age 14 onwards (21), we grouped our sample into younger children (< 14 years) and older children (≥14 years). Within the younger group of children (< 14) there were no differences when it comes to the number of girls (*n* = 164) and boys (*n* = 158) (χ^2^ = 0.112, df = 1, *p* > 0.05), nor age differences were observed between boys (*n* = 158, age, mean ± SD, 10.14 ± 2.23 years) and girls (*n* = 164, age, mean ± SD, 10.29 ± 2.52 years) (*t* = 5.78, df = 320, *p* > 0.05). However, within the older group of children (≥14 years), there were significantly more adolescent girls (*n* = 225) than boys (*n* = 101) (χ^2^ = 47.16, df = 1, *p* < 0.001), and girls were significantly older than boys (girls mean ± SD, 15.7 ± 1.15 years; boys mean ± SD, 15.3 ± 1.16 years; *t* = 2.72, df = 324, *p* = 0.003).

### Headache diagnosis

Overall, the mixed-type headache (any form of tension-type headache and episodic migraine with or without aura) (*n* = 164, 26.7%), together with episodic migraine without aura (*n* = 163, 26.5%) were the most prevalent headache diagnoses in our headache sample followed by chronic tension-type headache (*n* = 104, 16.9%). Chronic migraine (*n* = 9, 1.5%) was generally the least prevalent headache diagnosed in the overall patient sample, followed by other headaches (that included new daily persistent headache, vestibular migraine, trigeminal autonomic headaches, *n* = 38, 6.2%). Table 1 shows the distribution of the distinct headache diagnoses between girls and boys according to age group. In younger children (< 14 years), the mixed-type headache was the most prevalent diagnosis in girls (girls *n* = 45, 28.6%), whereas, for boys, episodic migraine without aura was the most prevalent headache diagnosis (boys *n* = 72, 47.4%). For adolescents (≥14 years), the mixed-type continued to be the most prevalent headache diagnosis for girls (*n* = 63, 29.9%), and it also became the most prevalent headache diagnosis for teenage boys (*n* = 25, 26.3%). Chronic migraine was the least common headache diagnosis for both girls and boys under (girls *n* = 2, 1.3%; boys *n* = 1, 0.7%) and above 14 years (girls *n* = 6, 2.8%; boys *n* = 0).

**Table 1:** Headache diagnosis by age group and sex (*n* = 615).

		**eMwoA**	**eMwA**	**CM**	**eTTH**	**cTTH**	**Mixed type**	**Other HD**	**Total**
**Age group**	**Sex**	***n* (%)**	***n* (%)**	***n* (%)**	***n* (%)**	***n* (%)**	***n* (%)**	***n* (%)**	***n* (%)**
< 14 years	Female	34 (21.7)	24 (15.3)	2 (1.3)	17 (10.8)	25 (15.9)	45 (28.6)	10 (6.4)	157 (50.8)
	Male	72 (47.4)	5 (3.3)	1 (0.7)	19 (12.5)	19 (12.5)	31 (20.4)	5 (3.3)	152 (49.2)
	Total	106 (34.3)	29 (9.4)	3 (1)	36 (11.7)	44 (14.2)	76 (24.6)	15 (4.9)	309 (50.2)
≥14 years	Female	34 (16.1)	22 (10.4)	6 (2.8)	24 (11.4)	48 (22.7)	63 (29.9)	14 (6.6)	211 (69)
	Male	23 (23.2)	16 (16.8)	0 (0)	10 (10.5)	12 (12.6)	25 (26.3)	9 (9.5)	95 (31)
	Total	57 (18.6)	38 (12.4)	6 (2)	34 (11.1)	60 (19.6)	88 (28.8)	23 (7.5)	306 (49.8)
All patients	Total	163 (26.5)	67 (10.9)	9 (1.5)	70 (11.4)	104 (16.9)	164 (26.7)	38 (6.2)	615 (100)

A significant relationship between sex, prevalence, age group, and headache diagnoses, was noted in the overall sample [χ^2^(1, *N* = 615) = 21.06, *p* < 0.001]. Within the younger group of children diagnosed with episodic migraine without aura, a significantly higher prevalence of boys (*n* = 72) as compared to girls (*n* = 34) (χ^2^ = 13.62, df = 1, *p* < 0.001) was observed. On the other hand, no significant differences were found in the distribution of teenage boys and girls with episodic migraine without aura (boys *n* = 23; girls *n* = 34; χ^2^ = 2.12, df = 1, *p* > 0.05). Regarding the mixed-type headache, no prevalence differences were found between girls and boys (girls *n* = 45; boys *n* = 31; χ^2^ = 2.57, df = 1, *p* > 0.05). Conversely, in the adolescent group with the mixed-headache type, there was a significantly higher prevalence of girls than boys (girls *n* = 63; boys *n* = 25; χ^2^ = 16.40; df = 1, *p* < 0.001).

Within the younger group of children with episodic migraine with aura, there were significantly more girls (*n* = 24) than boys (*n* = 5) (χ^2^ = 12.45, df = 1, *p* < 0.001). However, this difference dissipated within the teenage group (girls *n* = 22; boys *n* = 16; χ^2^ = 0.94; df = 1, *p* > 0.05). Among younger children with episodic tension headaches, there were no significant differences concerning sex distribution (girls *n* = 17; boys *n* = 19; χ^2^ = 0.11; df = 1, *p* > 0.05), whereas, among adolescents diagnosed with episodic tension headache, there was a significantly higher prevalence of girls (*n* = 24), than boys (*n* = 10) (χ^2^ = 5.76, df = 1, *p* < 0.016). Although in younger children with chronic tension headaches, no significant prevalence differences were observed among boys and girls (girls *n* = 25; boys *n* = 19; χ^2^ = 0.81; df = 1, *p* > 0.05), a significant difference in sex prevalence was observed in the adolescent group with chronic tension headaches (girls *n* = 48; boys *n* = 12; χ^2^ = 21.60; df = 1, *p* < 0.001).

### Headache related impairment

#### Headache days

**Figure 2:**
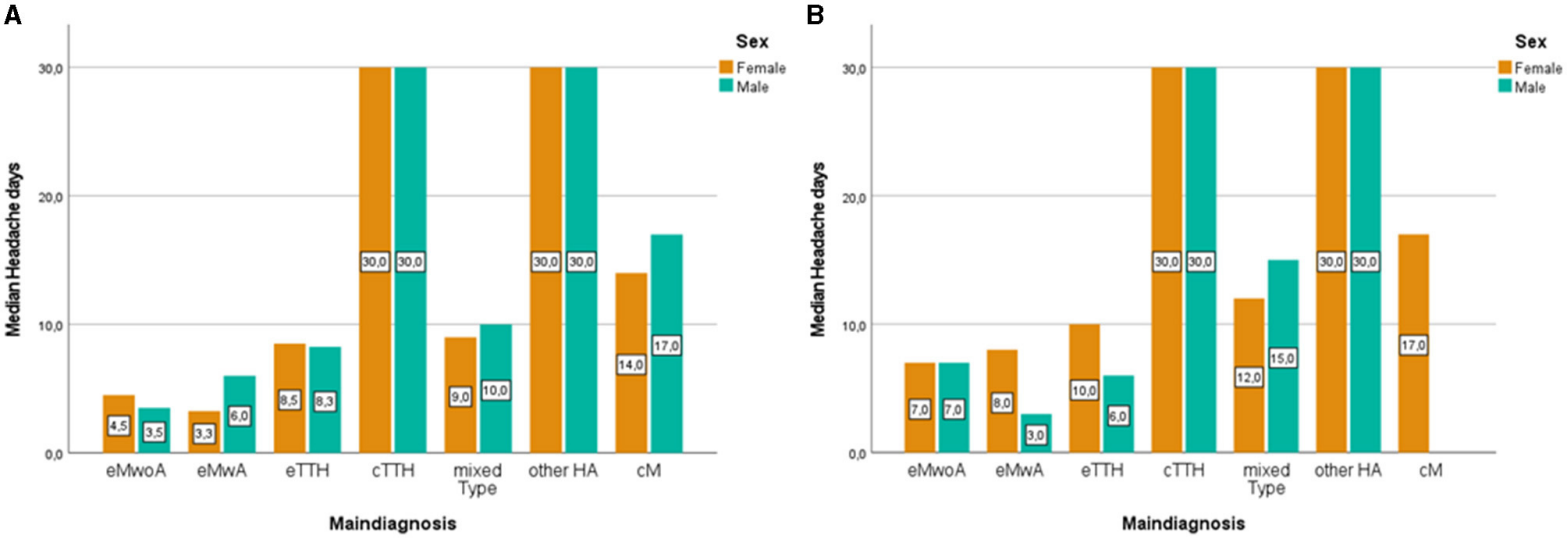
Clustered bar median of headache days in younger (<14) **(A)** and older children (≥14) **(B)** according to main diagnosis and sex. eMwoA, episodic Migraine without Aura; eMwA, episodic Migraine with Aura; CM, chronic Migraine; eTTH, episodic Tension-Type Headache; cTTH, chronic Tension-Type Headache; Mixed type, includes coexisting TTH and episodic Migraine; Other HA, other headaches.

During their initial visit, girls < 14 years [Median = 8, IQR (−32.2, 48.2)] reported significantly more headache days (*U* = 9,649, *N*_1_ = 153, *N*_2_ = 147, *p* = 0.033), during the last 3 months, as compared to boys in the same age group [Median = 6, IQR (−12, 24)]. Significant sex differences in reported headache days were also observed after the age of 14, with girls reporting again higher headache frequency as compared to boys [girls Median = 15, IQR (−18.75, 48.75); boys Median = 12, IQR (−27.0, 51.0); *U* = 7,761, *N*_1_ = 200, *N*_2_ = 93, *p* = 0.021]. Figure 2 illustrates headache frequency (3 months) considering sex, age group (a) < 14, *n* = 309; (b) ≥14, *n* = 306, and the distinct headache diagnosis. Before the age of 14 years, both girls [Median = 30, IQR (0)] and boys [Median = 30, IQR (3.0, 57)] with other headache disorders and chronic tension-type headaches displayed the highest headache frequency. The same pattern was observed in older children [girls Median = 30, IQR (23.13, 36.86); boys Median = 30, IQR (25.1, 34.9)]. No significant sex differences were found within the younger group of children, nor the older teenage group when it comes to reports of headache days within each distinct headache diagnosis (*p* > 0.05).

#### Headache intensity

**Figure 3:**
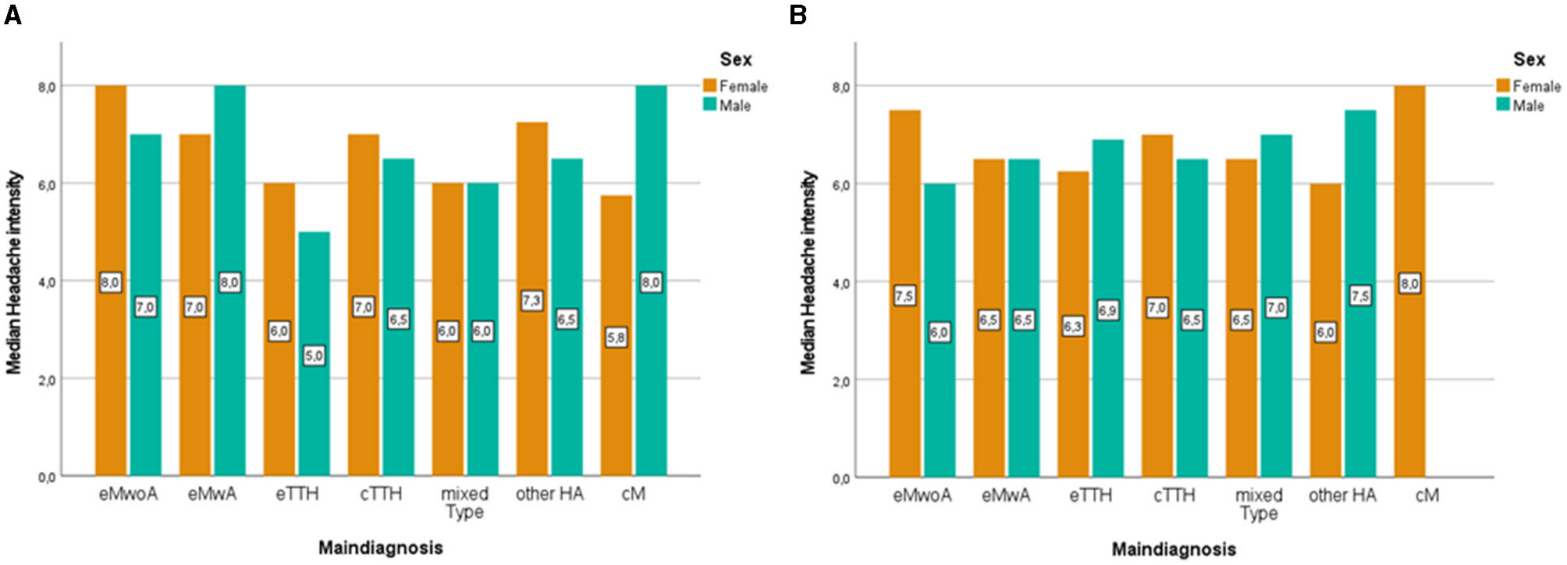
Clustered bar median of headache intensity in younger (<14) **(A)** and older children (≥14) **(B)** according to main diagnosis and sex. eMwoA, episodic Migraine without Aura; eMwA, episodic Migraine with Aura; CM, chronic Migraine; eTTH, episodic Tension-Type Headache; cTTH, chronic Tension-Type Headache; Mixed type, includes coexisting TTH and episodic Migraine; Other HA, other headaches.

When it comes to reports of headache intensity, there were no significant differences between girls and boys before [girls Median = 7, IQR (4.55, 9.45); boys Median = 6.5, IQR (3.56, 9.44); *U* = 9,979.5, *N*_1_ = 150, *N*_2_ = 144, *p* > 0.05] and after [girls Median = 7, IQR (4.55, 9.45); boys Median = 6.5, IQR (3.56, 9.44); *U* = 8,690, *N*_1_ = 200, *N*_2_ = 90, *p* > 0.05], the age of 14. Figure 3 illustrates headache intensity between boys and girls within distinct headache diagnoses in younger (a) and older (b) children. Younger boys (< 14 years) with chronic migraine and episodic migraine with aura [Median = 8, IQR (4.76, 11.24)] reported the highest headache intensity whereas in younger girls (< 14 years) episodic migraine without aura was described with the highest headache intensity [Median = 8, IQR (5.55, 10.45)]. In older girls, chronic migraine becomes the headache diagnosis with the highest headache intensity [Median = 8, IQR (4.57, 11.43)] whereas for older boys other headache disorders [Median = 7.5, IQR (3.78, 11.22)] were observed to have the highest reports of pain intensity. When examining sex differences, in reported headache intensity, within each specific headache diagnosis, the only significant differences observed were within young children with episodic migraine without aura (*U* = 788, *N*_1_ = 31, *N*_2_ = 68, *p* = 0.043) with girls reporting higher headache intensity levels [girls Median = 8, IQR (3.55, 8.45); boys Median = 7, IQR (4.55, 9.45)] no other significant sex differences were observed in either the younger or the older group of children.

### School absenteeism

**Figure 4:**
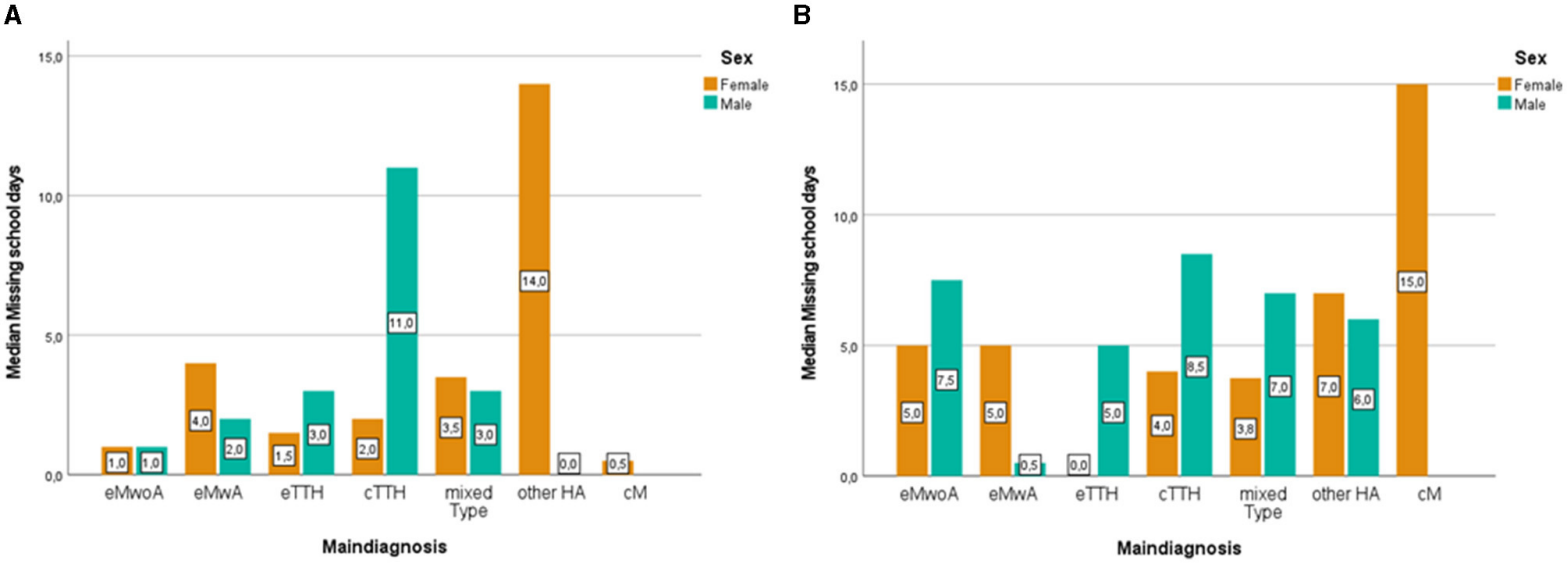
Clustered bar median of missing school days in the last 3 months in younger (<14) **(A)** and older children (≥14) **(B)** according to main diagnosis and sex. eMwoA, episodic Migraine without Aura; eMwA, episodic Migraine with Aura; CM, chronic Migraine; eTTH, episodic Tension-Type Headache; cTTH, chronic Tension-Type Headache; Mixed type, includes coexisting TTH and episodic Migraine; Other HA, other headaches.

With regards to school absenteeism due to headaches, during the past 3 months of their initial visit, our patients lost a mean of 6.9 ± 9.98 school days. Before the age of 14, there were no differences between girls [Median = 2, IQR (−4.86, 8.86)] and boys [Median = 2, IQR (−4.86, 8.86)] concerning school absenteeism due to headache (*U* = 9,766, *N*_1_ = 143, *N*_2_ = 139, *p* > 0.05). Likewise, after the age of 14, there were no significant differences between teenage boys [Median = 6, IQR (−7.72, 19.72)] and girls [Median = 5, IQR (−676, 16.76)] when it comes to missing schooldays (*U* = 7,104, *N*_1_ = 189, *N*_2_ = 84, *p* > 0.05). Figure 4 displays missing schooldays distribution according to age group, sex, and headache diagnose. Before the age of 14, school attendance seems to be mostly affected in girls with other headaches [Median = 14, IQR (−2.66, 30.66)], and in boys with chronic tension-type headaches [Median = 11, IQR (10.56, 32.56)]. In teenage boys, chronic tension-type headache continues to represent the biggest burden when it comes to missing school days [Median = 8.5, IQR (−22.28, 39.28)], whereas in teenage girls chronic migraine takes the lead in affecting school attendance [Median = 15, IQR (5.72, 24.28)]. While investigating sex differences in reported school absenteeism, results suggest that in older children with episodic migraine with aura, teenage girls miss significantly more school days as compared with teenage boys [girls Median = 5, IQR (−12.64, 22.64); boys Median = 0.5, IQR (−3.22, 4.22); *U* = 64, *N*_1_ = 19, *N*_2_ = 12, *p* = 0.043]. On the other hand, in younger children with chronic tension-type headaches, boys report higher levels of school absenteeism as compared to girls [boys Median = 11, IQR (−10.56, 32.56], girls Median = 2, IQR (−2.9, 6.9); *U* = 95, (_1_ = 21, *N*_2_ = 16, *p* = 0.025]. When it comes to young children with other headache diagnoses, results suggest that young girls report missing a higher number of school days as compared with young boys [girls Median = 14, IQR (2.66, 30.66), boys Median = 0, IQR (−7.35, 7.35); *U* = 4.50, *N*_1_ = 8, *N*_2_ = 14, *p* = 0.047].

### Headache disability

**Figure 5:**
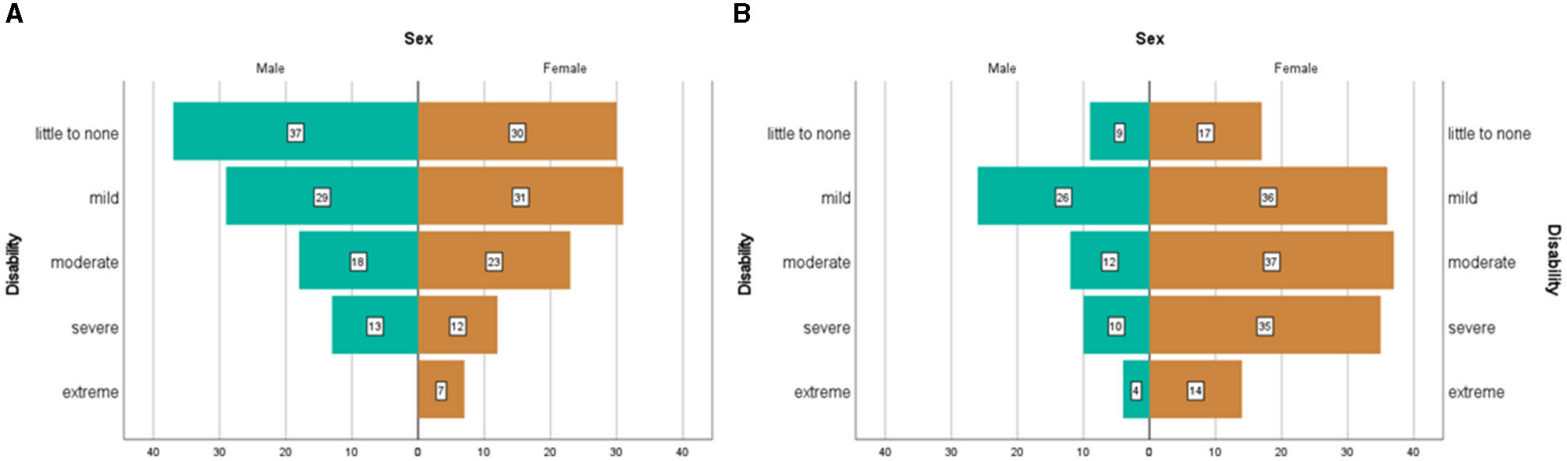
Distribution of the severity of headache disabilities or self-assessment questionnaire (13) in younger (<14) **(A)** and older children (≥14) **(B)** according to sex.

We found a significant relationship between sex, age group, and the level of headache disability, measured with PedMIDAS [χ^2^(1, *N* = 400) = 13.56, *p* < 0.001]. Figure 5 illustrates the disability reported by boys and girls within distinct age groups as a result of headaches. Whereas, before the age of 14, roughly one-third of children (34%) seemed to be less affected by their headaches and about 16% of children seem to be severely affected by their headaches. After the age of 14, the lives of roughly one-third of adolescents (33%) become severely or extremely affected by their headaches. This increase is especially driven by the percentage of teenage girls who become severely/extremely affected by their headaches (26%). Within the older female group, more than one-third of teenage girls (35.3%) were severely to extremely affected by headache disorders, whereas within the older male group less than one-fourth (23.0%) of teenage boys' lives were severely to extremely affected by headache disorders.

### Comorbidities

With regards to comorbidities, 44% of our patients (64% females) reported having at least one comorbidity. Notably, 27.6% (74.6% females) of these patients reported having at least 2 comorbidities and 5% (92.9% females) reported having three comorbidities. The prevalence of comorbidities was significantly higher among girls compared to boys (girls *n* = 184; boys *n* = 103; χ^2^ = 22.86; df = 1, *p* < 0.001). The overall sex difference is driven by the significant disparity observed in the adolescent group (girls *n* = 119; boys *n* = 48; χ^2^ = 30.19; df = 1, *p* < 0.001) a difference not evident in the younger group (girls *n* = 65; boys *n* = 54; χ^2^ = 1.017; df = 1, *p* > 0.05). Table 2 indicates the reported comorbidities by sex and age group (note that 27% of patients reported having more than one comorbidity). The most common comorbidity, with almost one-third of patients' reports, regardless of sex and age group, was internal disorders (30%), which include a wide spectrum of diseases. Within girls, the most commonly listed comorbidities were internal disorders (27.1%) followed by other pain disorders (25.1%) both under and above the age of 14. Among younger boys internal disorders were the most common reported comorbidities (42.8%) followed by neurological disorders (19%). In older boys, internal disorders (30%), were followed by mental disorders (20%). Within the older group of children, girls reported a significantly higher prevalence of internal disorders as compared to boys (χ^2^ = 9.600, df = 1, *p* = 0.002). A sex difference in the prevalence of internal disorders was not observed in the younger group (χ^2^ = 0.74, df = 1, *p* > 0.05). When it comes to other commonly reported comorbidities such as other pain disorders, a significantly higher prevalence was reported for both younger (χ^2^ = 6.760, df = 1, *p* = 0.009) and older (χ^2^ = 19.800, df = 1, *p* < 0.01) girls as compared to younger and older boys. In neurological disorders, no significant sex differences were found within the younger and older age groups (*p* > 0.05).

**Table 2:** Reported comorbidities by age group and sex (*n* = 378).

		**Internal**	**Mental**	**Orthopedic**	**Neurological**	**Other pain**	**Dermatological**	**Total**
**Age group**	**Sex**	***n* (%)**	***n* (%)**	***n* (%)**	***n* (%)**	***n* (%)**	***n* (%)**	***n* (%)**
< 14 years	Female	28 (32.9)	5 (5.9)	5 (5.9)	16 (18.8)	19 (22.3)	12 (14.1)	85 (57.4)
	Male	27 (42.8)	6 (9.5)	6 (9.5)	12 (19.0)	6 (9.5)	6 (9.5)	63 (42.6)
	Total	55 (37.2)	11 (7.4)	11 (7.4)	28 (18.9)	25 (16.9)	18 (12.2)	148 (39.2)
≥14 years	Female	41 (24.1)	34 (20.0)	20 (11.7)	21 (12.3)	45 (26.5)	9 (5.3)	170 (73.9)
	Male	18 (30.0)	12 (20.0)	7 (11.7)	11 (18.3)	11 (18.3)	1 (1.6)	60 (26.1)
	Total	59 (25.7)	46 (20)	27 (11.7)	32 (13.9)	56 (24.4)	10 (4.4)	230 (60.8)
Comorbidities	Total	114 (30.1)	57 (15.1)	38 (10.1)	60 (15.9)	81 (21.4)	28 (7.4)	378 (100)

## Discussion

In the present study, we considered age and sex while evaluating the distribution, characteristics, and impairment of primary headache disorders at a pediatric headache center in Germany.

### Sample characteristics and headache diagnosis by age group and sex

Almost two-thirds of 652 children and adolescents attending the headache clinic between 2015 and 2022 were females. After stratifying our headache population according to age and sex, we attested that the observed sex differences were driven by the adolescent (≥14) group and no sex differences were noted within the younger group. This goes in line with previous data suggesting that in childhood boys and girls are equally likely to be affected by primary headache disorders (15). In the adolescent group (≥14), girls were notably older than boys and significantly outnumbered adolescent boys, emulating adult headache prevalence ratios (22). These findings underscore the potential role of sex hormones in headache pathophysiology (23). During puberty, sexual steroid hormones have been shown to affect neural circuits and cause permanent changes in relevant brain areas such as the hypothalamus and the insula (24). Moreover, studies suggest that the onset of migraine typically coincides with the cyclic hormonal changes that begin around the time of menarche. Early onset of menarche seems to constitute a risk factor for the development of migraine (25). However, the contribution of sociocultural factors likely plays an additional role (26). In our sample, the mixed headache type (including any form of tension-type headache and episodic migraine with or without aura), together with the diagnosis of migraine without aura were the most prevalent headache diagnoses. Regarding the mixed-type headache, whereas no sex prevalence differences were found within the younger population (< 14 years), a significantly higher prevalence of girls was found in the adolescent group. This goes in line with adult studies showing that tension-type headaches, the most prevalent headache in clinical practice (27), and migraine have a female preponderance (28). This female preponderance has also been observed in persistent post-traumatic headache, predominantly characterized by somatic symptoms (29). Similar findings were observed in our tension-type headache diagnosis group, where a significantly higher prevalence of adolescent girls was observed. On the other hand, in episodic migraine without aura, a significantly higher prevalence of boys was noted within the younger group of children. This sex difference was no longer evident in the teenage group. This finding contrasts with the above-reported absence of sex prevalence differences within the younger group of headache disorders, emphasizing the importance of investigating distinct headache diagnoses separately. However, our results might represent the selection bias of a tertiary headache center or a sociocultural phenomenon, where migraine in girls tends not to be appreciated as a disease and therefore young girls are not enrolled for specific treatment.

Importantly, however, most headache literature evaluating the effect of sex in primary headaches comes from the migraine field, and there is a high need for research evaluating the variance of epidemiological patterns in distinct primary headache disorders by sex and age (10). This information holds the potential to advance our understanding of differential sex prevalence, and associated headache pathophysiology that in turn will guide the development of more successful therapies. For example, the onset of cluster headaches in women is often related to hormonal changes as milestones in biological development (30). Besides hormonal changes, behavioral and socioeconomic factors might influence sex-differences in headache prevalence (31).

Within our sample, chronic migraine emerged as the least prevalent among diagnosed headache disorders. This outcome likely mirrors deficient diagnostic criteria, with a considerable portion of children included in the mixed group potentially experiencing chronic migraine. In addition, it might be the result of fluctuations in migraine frequency in adolescents. A study of 2,517 migraine attacks in 115 children has shown a seasonal increase in migraine between November and January and a minimum in summer school vacation (32). This observation calls for a review of current diagnostic criteria for chronic migraine in children and adolescents. In line with this, the guidelines of the International Headache Society for controlled trials of preventive treatment of migraine in children and adolescents have been recently updated (33). Frequently starting in childhood and extending into adulthood (34), migraine is one of the highest specific cause of adult disability worldwide (1). Hence, early recognition and successful management of the disorder becomes vital.

### Headache-related impairment by age group and sex

Headache disorders are known to significantly impact children and adolescents' quality of life (8). In our headache sample, both younger and older girls reported having more headache days than younger and older boys. This pattern corroborates with literature suggesting that females are more affected than men, especially in their late teenage years (8). When considering the distinct headache diagnoses separately, no significant sex differences in reported headache days were observed in our younger group or our teenage group. This most likely reflects the insufficient sample size within distinct headache diagnoses or constitutes a selection bias of a tertiary headache center, where patients with higher headache frequencies are treated. Within our sample, children with other headache disorders and with chronic tension-type headaches displayed the highest headache frequency. This is expected and goes in line with the ICHD-III diagnostic criteria (18) for chronic tension-type headaches that request at least 15 headache days per month. The most common headache diagnosis in the group of other headaches is a new daily persistent headache, where per definition persistent daily headache is requested. When it comes to chronic tension-type headaches, results are in line with the latest report of the Global Burden of Disease study presenting staggering estimates of global disability-adjusted life years for chronic tension-type headaches (35). With regards to headache intensity and school absenteeism, no sex differences were observed in younger or older children in the overall headache sample. However, when considering the distinct diagnoses separately, results suggested that younger girls with episodic migraine without aura have higher headache intensity levels as compared to boys. Moreover, data suggests that within the younger population, school attendance was more affected in boys with chronic tension-type headaches than in girls. The scenario seems to be reversed for other headache disorders, with girls reporting more absenteeism than boys. In teenagers, girls with episodic migraine with aura had higher levels of school absenteeism than boys. However, teenage boys with chronic tension-type headaches reported higher levels of absenteeism than girls. Even though this data suggests the existence of differences within specific headache disorders, our samples in some of these groups are very limited, which restrains robust inferences and underscores the need for further studies to delve into this matter with greater certainty provided by more extensive and diverse sample sizes. Looking at the overall pattern of headache-related disability, whereas before the age of 14, only about 16% of children seemed to be severely affected by their headaches, after the age of 14, the lives of roughly one-third of the adolescents were severely or extremely affected by their headaches. This increase seemed to be particularly driven by the percentage of teenage girls who were severely/extremely affected by their headaches. More than one-third of teenage girls (35.3%) over the age of 14 were severely to extremely affected by headache disorders. These findings are in line with recent data suggesting that the burden of headache disorders after the age of 15 is severe, particularly among females (36).

### Comorbidities by sex and age group

Headache disorders are commonly associated with several comorbidities, which substantially add to the global burden of headaches (37). In the present study, almost half of the pediatric patients reported having at least one comorbidity, which constitutes a high additional burden for the pediatric population. Notably, however, the prevalence of reported comorbidities was higher among girls than boys, particularly in the older group of patients, further supporting the literature showing a higher burden of headache disorders for females. In our pediatric sample, internal disorders (encompassing a broad spectrum of diseases) were the most commonly reported comorbidities. These results, however, were mostly driven by teenage girls. When it comes to pain disorders, the second most common comorbidity in our sample, again girls reported significantly more comorbid pain disorders than boys. Our findings provide additional evidence supporting the impact of sex and age when it comes to the distribution and trends of headache burden, particularly in later teenage years where girls seem to bear a greater burden of headache disorders (10). However, it is worth noting that the burden of headache disorders has been increasing over the past decades for teenage boys (36). Overall the presence of comorbidities adds complexity to the clinical management and outcomes of primary headaches. Understanding the mechanisms leading to these comorbid conditions remains challenging. Comorbidities can function as risk factors for the chronicity of headaches and as triggers for headache episodes (38). They might result from repeated headache attacks, treatments, or other shared factors, contributing to the progression of headaches into chronic forms. Besides migraine, there is a notable gap in the understanding of other headache-diagnosis-related comorbidities. Exploring the relationships between primary headache disorders and specific comorbidities can offer valuable epidemiological and clinical insights guiding accurate diagnoses and effective treatments.

### Limitations

Our disorder-specific sub-analysis is suggestive of age and sex differences within specific headache disorders, however, limited sample sizes in some subgroups significantly constrain robust inferences. This highlights the importance of large-scale research in the area (more extensive and diverse sample sizes). Due to our limited sample size, especially when it comes to the stratification of distinct headache diagnoses, our study does not assess the relationship between specific headache disorders and comorbidities, a critical avenue that has the potential to inform both pediatric diagnosis and pediatric treatment strategies. The lack of detailed recording of menarche variability, is an important factor to consider in future studies. Furthermore, the variability in subject numbers for our analyses, driven by the uneven availability of data, stands as a limitation. Yet, this discrepancy serves to echo the complexities encountered in the clinical realm, providing a realistic portrayal of the clinical challenges. Another limitation is that the data was collected from a single specialized center, which may affect the generalizability of the results to a broader population. In our study, there were no unclassified cases, which are commonly observed in other studies using different diagnostic methodologies (39). Additionally, the retrospective nature of the study may introduce biases due to the reliance on existing records. Despite this limitation, retrospective reviews of medical records remain a widely used and invaluable method in clinical research, facilitating the extraction of significant insights from large clinical datasets. Finally, in our study, about 27% of patients presented with symptoms characteristic of both migraine and TTH, meeting the diagnostic criteria for both conditions. This dual diagnosis highlights the clinical challenges in differentiating between these headache types in pediatric populations (17, 40), reflecting the need for refined diagnostic approaches.

## Conclusion

Overall, our data supports the assertion that headache disorders impose a significant burden, especially among teenage girls. The sex difference remains a work assignment, and additional efforts are needed to study headache disorders beyond migraine, in children and adolescents. Enhancing awareness of early diagnosis and preventive therapies is crucial to mitigate the development of chronic headaches and their adverse effects on life quality. Despite advancements in migraine management, the strong inequalities in care persist globally, exacerbated by socioeconomic disparities, limited access to novel therapies, and the ongoing impacts of the COVID-19 pandemic (41, 42). Future work should assess the feasibility and long-term tolerability of implementing successful therapies globally. Additionally, awareness campaigns and institutionalized education on headaches in schools are needed to combat stigma and misconceptions about migraines.

## Data availability statement

The raw data supporting the conclusions of this article will be made available by the authors, without undue reservation.

## Ethics statement

Retrospective data analysis was approved by the Ethics Committee of the TU Dresden (GVOEK) under the application number EK 264062020. Written informed consent to participate in this study was not required from the participants or the participants' legal guardians/next of kin in accordance with the national legislation and the institutional requirements.

## Author contributions

VF: Writing – review & editing, Writing – original draft, Validation, Methodology, Investigation, Formal analysis, Data curation, Conceptualization. BH: Writing – review & editing, Validation, Methodology, Formal analysis, Conceptualization. AK: Writing – review & editing, Validation, Methodology, Formal analysis, Data curation. MH: Writing – review & editing, Validation, Supervision, Methodology, Investigation, Formal analysis. RB: Writing – review & editing, Validation, Supervision, Methodology. RS: Writing – review & editing, Validation, Supervision, Methodology, Investigation. TK: Writing – review & editing, Validation, Supervision, Methodology, Investigation. AH: Writing – review & editing, Validation, Supervision, Methodology, Investigation. MR: Writing – review & editing, Validation, Supervision, Methodology, Investigation. EM: Writing – review & editing, Validation, Supervision, Methodology. SH: Writing – review & editing, Validation, Supervision, Methodology, Investigation. GG: Writing – review & editing, Validation, Supervision, Project administration, Methodology, Investigation, Funding acquisition, Data curation, Conceptualization.

## References

[1] GBD 2016 Headache Collaborators. Global, regional, and national burden of migraine and tension-type headache, 1990-2016: a systematic analysis for the Global Burden of Disease Study 2016. Lancet Neurol. (2018) 17:954–76. 10.1016/S1474-4422(18)30322-3 30353868 PMC6191530

[2] Steiner TJ Birbeck GL Jensen RH Katsarava Z Stovner LJ Martelletti P. Headache disorders are third cause of disability worldwide. J Headache Pain. (2015) 16:58. 10.1186/s10194-015-0544-2 26109437 PMC4480232

[3] GBD 2016 Disease and Injury Incidence and Prevalence Collaborators. Global, regional, and national incidence, prevalence, and years lived with disability for 328 diseases and injuries for 195 countries, 1990-2016: a systematic analysis for the Global Burden of Disease Study 2016. Lancet. (2017) 390:1211–59. 10.1016/S0140-6736(17)32154-2 28919117 PMC5605509

[4] Steiner TJ Stovner LJ Katsarava Z Lainez JM Lampl C Lantéri-Minet M. The impact of headache in Europe: principal results of the Eurolight project. J Headache Pain. (2014) 15:31. 10.1186/1129-2377-15-31 24884549 PMC4045992

[5] Seddik AH Branner JC Ostwald DA Schramm SH Bierbaum M Katsarava Z. The socioeconomic burden of migraine: an evaluation of productivity losses due to migraine headaches based on a population study in Germany. Cephalalgia. (2020) 40:1551–60. 10.1177/0333102420944842 32762249

[6] Wöber-Bingöl Ç. Epidemiology of migraine and headache in children and adolescents. Curr Pain Headache Rep. (2013) 17:341. 10.1007/s11916-013-0341-z 23700075

[7] Al-Hashel JY Ahmed SF Alroughani R. Prevalence and burden of primary headache disorders in Kuwaiti children and adolescents: a community based study. Front Neurol. (2019) 10:793. 10.3389/fneur.2019.00793 31417482 PMC6682654

[8] Leonardi M Grazzi L D'Amico D Martelletti P Guastafierro E Toppo C. Global burden of headache disorders in children and adolescents 2007-2017. Int J Environ Res Public Health. (2020) 18:250. 10.3390/ijerph18010250 33396281 PMC7795582

[9] Goodman JE McGrath PJ. The epidemiology of pain in children and adolescents: a review. Pain. (1991) 46:247–64. 10.1016/0304-3959(91)90108-A 1758709

[10] Onofri A Pensato U Rosignoli C Wells-Gatnik W Stanyer E Ornello R. Primary headache epidemiology in children and adolescents: a systematic review and meta-analysis. J Headache Pain. (2023) 24:8. 10.1186/s10194-023-01541-0 36782182 PMC9926688

[11] Poyrazoglu HG Kumandas S Canpolat M Gümüs H Elmali F Kara A. The prevalence of migraine and tension-type headache among schoolchildren in Kayseri, Turkey: an evaluation of sensitivity and specificity using multivariate analysis. J Child Neurol. (2015) 30:889–95. 10.1177/0883073814549240 25296924

[12] Krogh A-B Larsson B Linde M. Prevalence and disability of headache among Norwegian adolescents: a cross-sectional school-based study. Cephalalgia. (2015) 35:1181–91. 10.1177/0333102415573512 25720767

[13] Nieswand V Richter M Berner R Hagen Mv Klimova A Roeder I. The prevalence of headache in German pupils of different ages and school types. Cephalalgia. (2019) 39:1030–40. 10.1177/0333102419837156 30884960

[14] Barea LM Tannhauser M Rotta NT. An epidemiologic study of headache among children and adolescents of southern Brazil. Cephalalgia. (1996) 16:545–9; discussion 523. 10.1046/j.1468-2982.1996.1608545.x 8980856

[15] Ursitti F Valeriani M. Migraine in childhood: gender differences. Eur J Paediatr Neurol. (2023) 42:122–5. 10.1016/j.ejpn.2023.01.002 36634526

[16] Straube A Andreou A. Primary headaches during lifespan. J Headache Pain. (2019) 20:35. 10.1186/s10194-019-0985-0 30961531 PMC6734460

[17] Baglioni V Orecchio S Esposito D Faedda N Natalucci G Guidetti V. Tension-type headache in children and adolescents. Life. (2023) 13:825. 10.3390/life13030825 36983980 PMC10056425

[18] Headache Headache Classification Committee of the International Headache Society (IHS) The International Classification of Headache Disorders 3rd edition. Cephalalgia. (2018) 38:1–211. 10.1177/0333102417738202 29368949

[19] Nieswand V Richter M Gossrau G. Epidemiology of headache in children and adolescents-another type of pandemia. Curr Pain Headache Rep. (2020) 24:62. 10.1007/s11916-020-00892-6 32840694 PMC7447651

[20] Burch R Rizzoli P Loder E. The prevalence and impact of migraine and severe headache in the United States: updated age, sex, and socioeconomic-specific estimates from government health surveys. Headache. (2021) 61:60–8. 10.1111/head.14024 33349955

[21] Albers L Kries RV Straube A Heinen F Landgraf MN Obermeier V. Age- and sex-specific first health care use for migraine in 2016 in children and adolescents from prospectively collected health insurance data in Germany. Cephalalgia. (2019) 39:1156–63. 10.1177/0333102419844543 30974953

[22] Stovner LJ Hagen K Linde M Steiner TJ. The global prevalence of headache: an update, with analysis of the influences of methodological factors on prevalence estimates. J Headache Pain. (2022) 23:34. 10.1186/s10194-022-01402-2 35410119 PMC9004186

[23] Gupta S Mehrotra S Villalón CM Perusquía M Saxena PR MaassenVanDenBrink A. Potential role of female sex hormones in the pathophysiology of migraine. Pharmacol Ther. (2007) 113:321–40. 10.1016/j.pharmthera.2006.08.009 17069890

[24] Borsook D Erpelding N Lebel A Linnman C Veggeberg R Grant PE. Sex and the migraine brain. Neurobiol Dis. (2014) 68:200–14. 10.1016/j.nbd.2014.03.008 24662368 PMC4171725

[25] Maleki N Kurth T Field AE. Age at menarche and risk of developing migraine or non-migraine headaches by young adulthood: a prospective cohort study. Cephalalgia. (2017) 37:1257–63. 10.1177/0333102416677999 27919016

[26] Ge R Chang J Cao Y. Headache disorders and relevant sex and socioeconomic patterns in adolescents and young adults across 204 countries and territories: an updated global analysis. J Headache Pain. (2023) 24:110. 10.1186/s10194-023-01648-4 37592213 PMC10436621

[27] Fan X Fu G Wang L Shen W Zhang Y. A bibliometric analysis and visualization of tension-type headache. Front Neurol. (2022) 13:980096. 10.3389/fneur.2022.980096 36119709 PMC9471986

[28] Vetvik KG MacGregor EA. Sex differences in the epidemiology, clinical features, and pathophysiology of migraine. Lancet Neurol. (2017) 16:76–87. 10.1016/S1474-4422(16)30293-9 27836433

[29] McEvoy H Borsook D Holmes SA. Clinical features and sex differences in pediatric post-traumatic headache: a retrospective chart review at a Boston area concussion clinic. Cephalalgia. (2020) 40:701–11. 10.1177/0333102419896754 31865762

[30] Allena M De Icco R Sances G Ahmad L Putortì A Pucci E. Gender differences in the clinical presentation of cluster headache: a role for sexual hormones? Front Neurol. (2019) 10:1220. 10.3389/fneur.2019.01220 31824403 PMC6882735

[31] Zimmer Z Fraser K Grol-Prokopczyk H Zajacova A. A global study of pain prevalence across 52 countries: examining the role of country-level contextual factors. Pain. (2022) 163:1740–50. 10.1097/j.pain.0000000000002557 35027516 PMC9198107

[32] Soriani S Fiumana E Manfredini R Boari B Battistella PA Canetta E. Circadian and seasonal variation of migraine attacks in children. Headache. (2006) 46:1571–4. 10.1111/j.1526-4610.2006.00613.x 17115990

[33] Abu-Arafeh I Hershey AD Diener H-C Tassorelli C. Guidelines Update: Guidelines of the International Headache Society for controlled trials of preventive treatment of migraine in children and adolescents, 1st edition - An experience-based update. Cephalalgia. (2023) 43:3331024231178239. 10.1177/03331024231178239 37226450

[34] Waliszewska-Prosół M Straburzyński M Czapińska-Ciepiela EK Nowaczewska M Gryglas-Dworak A Budrewicz S. Migraine symptoms, healthcare resources utilization and disease burden in a large Polish migraine cohort: results from 'Migraine in Poland'-a nationwide cross-sectional survey. J Headache Pain. (2023) 24:40. 10.1186/s10194-023-01575-4 37041492 PMC10091674

[35] Steel SJ Robertson CE Whealy MA. Current understanding of the pathophysiology and approach to tension-type headache. Curr Neurol Neurosci Rep. (2021) 21:56. 10.1007/s11910-021-01138-7 34599406

[36] Yuan R Tong Z Xiang G Xie Y Li K Zhang L. The burden and trends of headache disorders among the population aged 15–39: a study from 1990 to 2019. J Headache Pain. (2023) 24:168. 10.1186/s10194-023-01703-0

[37] Caponnetto V Deodato M Robotti M Koutsokera M Pozzilli V Galati C. Comorbidities of primary headache disorders: a literature review with meta-analysis. J Headache Pain. (2021) 22:71. 10.1186/s10194-021-01281-z 34261435 PMC8278743

[38] Buse DC Reed ML Fanning KM Bostic R Dodick DW Schwedt TJ. Comorbid and co-occurring conditions in migraine and associated risk of increasing headache pain intensity and headache frequency: results of the migraine in America symptoms and treatment (MAST) study. J Headache Pain. (2020) 21:23. 10.1186/s10194-020-1084-y 32122324 PMC7053108

[39] Wöber-Bingöl C Wöber C Karwautz A Vesely C Wagner-Ennsgraber C Amminger GP. Diagnosis of headache in childhood and adolescence: a study in 437 patients. Cephalalgia. (1995) 15:13–21; discussion 4. 10.1046/j.1468-2982.1995.1501013.x 7758092

[40] Genizi J Matar AK Schertz M Zelnik N Srugo I. Pediatric mixed headache -The relationship between migraine, tension-type headache and learning disabilities - in a clinic-based sample. J Headache Pain. (2016) 17:42. 10.1186/s10194-016-0625-x 27102119 PMC4840135

[41] Tana C Raffaelli B Souza MNP de la Torre ER Massi DG Kisani N. Health equity, care access and quality in headache – part 1. J Headache Pain. (2024) 25:12. 10.1186/s10194-024-01712-7 38281917 PMC10823691

[42] Ashina M Katsarava Z Do TP Buse DC Pozo-Rosich P Özge A. Migraine: epidemiology and systems of care. Lancet. (2021) 397:1485–95. 10.1016/S0140-6736(20)32160-7 33773613

